# Measles vaccine coverage and factors related to uncompleted vaccination among 18-month-old and 36-month-old children in Kyoto, Japan

**DOI:** 10.1186/1471-2458-5-59

**Published:** 2005-06-04

**Authors:** Takayo Matsumura, Takeo Nakayama, Shigeru Okamoto, Hideko Ito

**Affiliations:** 1 Department of Health Informatics, Kyoto University School of Public Health, Kyoto, Japan; 2 Kami-gyo Public Health Center, City of Kyoto, Kyoto, Japan; 3 Department of General Medicine and Clinical Epidemiology, Kyoto University Graduate School of Medicine, Kyoto, Japan; 4 Fushimi Public Health Center, City of Kyoto, Kyoto, Japan

## Abstract

**Background:**

Due to low vaccine coverage, Japan has not only experienced outbreaks of measles but has also been exporting it overseas. This study aims to survey measles vaccine coverage and the factors uncompleted vaccination among community-living children.

**Methods:**

Subjects were the parents whose children had undergone either an 18-month or a 36-month checkup publicly provided by Kyoto City during November 2001 to January 2002. An anonymous self-administered questionnaire survey was conducted.

**Results:**

The coverage was 73.2% among the 18-month-old children (n = 2707) and 88.9% among the 36-month-old children (n = 2340), respectively. The following characteristics of mothers were related to uncompleted measles vaccination: aged below 30, working, concerned about the adverse events of the vaccine, and had insufficient knowledge. Similarly, the following characteristics among children were related to uncompleted measles vaccination: not the first-born child, interacting with other children in group settings. The coverage was the lowest among the children whose mothers were concerned about the adverse events of the vaccine without proper knowledge of measles and its vaccination.

**Conclusion:**

To increase vaccine coverage among children, parents' awareness about measles and vaccination against it should be promoted, especially for working mothers. Efforts to enhance access to vaccination services and to communicate with parents about changing vaccination schedules are necessary.

## Background

Despite the existence of a safe, effective, and inexpensive vaccine, measles is still not being controlled in many parts of the world. The use of measles vaccine over the last 30 years has reduced global measles morbidity and mortality by 74 and 85%, respectively, compared with the prevaccine era. Regional elimination goals have been established in the Americas (by 2000), Europe (by 2007) and the Eastern Mediterranean (by 2010) [1].

Vaccine coverage in excess of 95% interrupts endemic transmission of measles in many countries, but achievement of such coverage almost always requires great collaborative efforts. [2] The global strategy began in the 1990s and many countries implemented immunization and enhanced measles surveillance policies related to this during the late 1990's. Some (e.g. the Americas) achieved measles elimination quite early [2,3]. Since 2000, the WHO and UNICEF have recommended that, in addition to achieving high coverage with the first dose of measles vaccine, all children should be offered a second opportunity for measles vaccination to increase the total number of people who are immune [4].

In the United States, measles was targeted for elimination, and persisted at low incidence until 1989, when an epidemic swept the country. To prevent spread among school-age populations, a second dose of MMR vaccine is recommended. To increase immunization coverage among inner-city preschool populations, a number of activities have been undertaken to improve the immunization delivery system[5,6]. Today, measles has been eliminated in the United States [7].

During 1997 and 2001, a total of 156 (82%) of 191 countries provided a second opportunity to receive a vaccination through supplementary immunization activities or through routine health services. Japan remains as one of the remaining 35 countries that do not give a second opportunity for measles vaccination [4]. Of the 26 imported measles cases detected in the US in 2000, the largest number of these cases was from Japan (seven cases). In 2000, Japan is reported to be the principal exporting country of measles to the US [7].

The Immunization Law in Japan has been providing children with measles vaccination since 1978. Since Measles-mumps-rubella (MMR) vaccine was introduced into Japan in 1989, a number of cases of post-vaccination aseptic meningitis have been reported and these have been attributed to the use of Urabe Am9 mumps vaccine [8]. In 1993, the Ministry of Health and Welfare (MHW) withdrew the domestically produced MMR vaccine [9]. As of 1994, an amendment to the Immunization Law made vaccination voluntary and not mandatory. According to the present law, a single dose of measles vaccine is recommended for children over one year of age. Children are eligible to receive measles vaccination after 12 months following birth but not beyond 90 months. Until January 2004, adminisiration of measles vaccine was recommended between 12 and 24 months of age, instead of between 12 and 15 months when children have the greatest risk of contracting measles [10]. In Japan, measles vaccine coverage has remained low, and either small or moderate outbreaks have occurred repeatedly in communities. According to an infectious disease surveillance (2000), total measles cases were estimated to be from 180,000 to 210,000, and total deaths were estimated to be 88 [11,12]. Measles cases are most frequently observed among non-immunized children, particularly between 12 to 24 months. However, a nation-wide survey conducted in 2000 showed that measles vaccine coverage in Japan was 81.4%, which is not enough to prevent outbreaks [13]. In this context, the Japan Pediatric Association, the Japan Child Health Association, and the Japanese Association of Pediatrics jointly appealed to the Ministry of Health, Labor and Welfare to promote vaccination against measles in July 2001 [14]. Although secondary vaccine failure (SVF) has increased in Japan and discussion on two dose measles immunization has begun [15,16], actual increase in vaccine coverage among children between 12 to 24 months-old become a priority in the present setting. The level of vaccine coverage within a community is necessary to develop appropriate measures. Still, no public system in Japan is capable of providing such information on a regular basis.

The purpose of the present study is to examine the following: measles vaccine coverage among 18-month-old and 36-month-old children; parents' knowledge of and perceptions about measles and its vaccination; and factors related to uncompleted measles vaccination in Japanese local communities.

## Methods

The present survey was conducted in Kyoto City, an area that covers 610 km^2 ^and is situated in the middle of the Kinki district of Japan and has a population of 1.47 million as of October 2001. The number of one-year-olds and three-year-olds in Kyoto City are approximately 12,000 respectively. The survey covers a period of three months extending from November 2001 to January 2002. Subjects were parents of children who had received either an 18-month or a 36-month checkup at either one of the 11 health centers or the three substations in Kyoto City. In Japan, parents are encouraged to have their children receive 18-month and 36-month checkups at public health centers. The present survey was conducted in cooperation with the Kami-gyo Public Health Center located in Kyoto City. An anonymous self-administered questionnaire was sent out to each subject, enclosed with a pre-consultation form, which is usually posted to the parents prior to the health checkup. Questionnaire, which subjects completed at home, were collected on the day of the checkup. The questionnaire included items on relevant attributes of parents (e.g. gender, age, job status); relevant attributes of children (e.g. birth order, whether or not they interacted with other children in group settings (e.g. attending nursery schools), and presence/absence of allergy); the child's vaccination history; whether or not the child had contracted measles in the past; reasons for not receiving measles vaccination (if the relevant child had not been vaccinated against measles); knowledge regarding measles and its vaccination, and sources of information regarding measles vaccination.

Questionnaire items regarding knowledge of measles and its vaccination were developed based on a survey conducted in 1991, which investigated parents' awareness of the plausible grave consequences of contracting measles and the importance of measles vaccination [17].

This study's outcome measure is defined as measles vaccine coverage among 18 – and 36-month-old children. A logistic regression analysis was carried out to assess the factors related to uncompleted measles vaccination. To investigate how different levels of knowledge regarding measles and its vaccination, and varying degrees of concern for possible adverse events could influence uncompleted measles vaccination, the respondents were categorized into four groups according to their levels of knowledge and degree of concern. Respondents were also divided into a "low knowledge" group and a "high knowledge" group based on the median split of their total scores on a set of questions regarding knowledge of measles and its vaccination. A score of 0–6 points among the 18-month-old group and a score of 0–5 points among the 36-month-old group was deemed as indicative of "low knowledge", while a score of 7–12 points among the 18-month-old group and a score of 6–12 points among the 36-month-old group was deemed as indicative of "high knowledge." Results of the logistic regression analysis carried out using three dummy variables, namely X1, X2 and X3, are shown in Table 1.

**Table 1:** Characteristics of group 1~4

	knowledge	concern	dummy variable	Number	
				18-month group	36-month group
Group 1	high	Low		690	748
Group 2	low	Low	X1	1145	813
Group 3	high	high	X2	367	379
Group 4	low	high	X3	418	306

Statistical software (JMP4J, SAS institute) was used to carry out the analysis. For the validation study, the subject's answer to the question regarding the child's vaccination history was checked against the child's Personal Health Record Book (this control was conducted only for a certain number of subjects).

## Results

### The number of children who received a health checkup and the response rate

During the survey period, 90.6% of 18-month-old children (3072 out of 3391) and 85.7% of 36-month-old children (2836 out of 3308) in the city received a health checkup. The response rate was 88.1% (2707 respondents out of 3072 patients who received a health checkup) and 82.5% (2340 respondents out of 2836 patients who received a health checkup). Because the majority of parents who responded to the questionnaire were mothers (98.1% of respondents among the 18-month-old group and 99.8% of respondents among the 36-month-old group), the analysis was conducted under the assumption that all respondents were mothers.

### Measles vaccine coverage and incidence of measles

Measles vaccine coverage was calculated by dividing number of respondents whose children had been vaccinated against measles by total number of respondents. Coverage was 73.2% among the 18-month-old group and 88.9% among the 36-month-old group. In both groups, there were 44 children with a history of measles. The cumulative incidence of measles was calculated as 1.6% for the 18-month-old group and 1.9% for the 36-month-old group. In both groups, measles incidence was significantly higher among children who interacted with other children in group settings compared to children who did not. (18-month-old group: 3.3% (95%CI: 2.1–5.1) vs. 1.2% (95%CI: 0.8–1.7), 36-month-old group: 2.9% (95%CI: 2.0–4.3) vs. 1.3% (95%CI: 0.8–2.0))

To test validity, we compared responses about their children's history of measles vaccination from all respondents whose children received a health checkup at Kami-gyo Public Health Center during this survey period with the data registered in their children's Personal Health Record Books. Results showed that 98.2% of respondents (108 out of 110 respondents) in the 18-month-old group and 98.3% of respondents (117 out of 119 respondents) in the 36-month-old group provided valid answers regarding their children's history of measles vaccination.

### The reasons for not receiving measles vaccine

Respondents whose children had not been vaccinated against measles (698 18-month-old children and 211 36-month-old children) were asked about the reasons for not having their child be vaccinated. (Table 2) In both groups, the majority of respondents replied that either they had not received vaccination but were planning to in the near future or that they had wanted to receive vaccination but had missed the opportunity to do so. The percentage of respondents who indicated concern about adverse events from a vaccination was 2.9% among the 18-month-old group and 9.5% among the 36-month-old group, with the percentage being slightly higher for the latter group.

**Table 2:** Reasons for not receiving measles vaccination (multiple answers)

	18-month-old group	36-month-old group
Number of non-vaccinated children	n = 698	n = 211
	%	%
has not yet received measles vaccination but is going to receive it in the near future	76.2	46.9
had a cold at the time of vaccination	32.5	24.2
had to receive other vaccinations first	23.5	11.4
wanted to receive measles vaccination but missed the chance to receive it	22.2	34.1
wasn't aware of/forgot about the vaccination schedule	6.9	9.5
was sick at the time of vaccination	4.9	1.9
has already been infected with measles	4.9	11.8
was concerned about adverse events of measles vaccination	2.9	9.5
naturally acquired immunity seems more effective than artificially acquired immunity	2.4	4.7
vaccination is no longer mandatory	2.4	6.6
vaccination does not seem to be sufficiently effective	0.7	1.4
Others	5.4	3.3

### Knowledge about measles

The percentage of respondents who were aware that all children should be vaccinated against measles once they have reached the age of one was 78.7% among the 18-month-old group and 71.7% among the 36-month-old group. While approximately half of respondents were aware that measles is a highly contagious disease with symptoms of fever and rashes, only about 30% were aware that there have been cases of death from measles in Japan or that measles-infected children can suffer from the sequelae of measles. (Table 3) There were a total of 12 questions regarding knowledge of measles, with one point assigned to each question. The median of the total score for the 12 questions were 6 points in the 18-month-old group and 5 points in the 36-month-old group.

**Table 3:** Knowledge about measles among the mothers of children eligible for 18-month or 36-month checkup

	Percentage of respondents answering "yes"	
	
	18-month group	36-month group
Participants	n = 2707	n = 2340
All children should be vaccinated against measles once they have reached the age of one	78.7	71.7
Measles is accompanied by high fever	74.1	76.2
Measles is accompanied by rashes	72.6	73.7
Measles is highly contagious	66.4	67.9
Most people can be immunized against measles through vaccination	50.4	51.0
The best way to prevent measles is through vaccination	45.1	45.7
Severe cases of measles are more likely to occur in younger children	41.6	34.4
There are some reported cases of death from measles in Japan	40.7	37.6
Measles can cause encephalitis	37.6	38.4
Some measles-infected children may suffer from the sequelae of measles	30.7	30.7
Measles can cause pneumonia	26.0	24.0
Measles is accompanied by severe coughing	10.0	8.8

### Predictors of uncompleted measles vaccination

A logistic regression analysis was conducted considering the following variables: mother's age (< 30 years), mother working, not first born child, interaction with other children in group settings, presence of allergies in child, mother having concern about the adverse events of measles vaccination, "low knowledge" about measles. (Table 4) Respondents were divided into a "low knowledge" group and a "high knowledge" group based on the median split of their scores on a set of questionnaire items related to the knowledge of measles and its vaccination. Among the variables related to mothers' characteristics, working and concern about adverse events of measles vaccination, and "low knowledge" of measles were significantly associated with uncompleted measles vaccination. Among variables related to child's characteristics, not first born child and interaction with other children were significantly associated with uncompleted measles vaccination.

**Table 4:** Factors related to uncompleted measles vaccination (univariate analysis)

	variable	Prevalence (%)	Odds Ratio	(95% CI)	P value
18-month group	Mother's age (< 30 years)	36.7	1.11	(0.93, 1.32)	0.26
	Mother working	25.1	1.66	(1.37, 2.01)	<0.0001
	Not first born child	45.5	1.93	(1.62, 2.30)	<0.0001
	Interaction with other children	21.2	1.76	(1.44, 2.15)	<0.0001
	Presence of allergy	17.8	0.90	(0.71, 1.13)	0.37
	Concern about adverse events	30.0	1.30	(1.07, 1.56)	0.007
	Low knowledge	60.7	2.09	(1.73, 2.53)	<0.0001

36-month group	Mother's age (< 30 years)	22.5	1.32	(0.95, 1.81)	0.09
	Mother working	33.3	2.60	(1.95, 3.49)	<0.0001
	Not first born child	46.5	2.04	(1.53, 2.74)	<0.0001
	Interaction with other children	38.3	2.64	(1.98, 3.54)	<0.0001
	Presence of allergy	27.9	1.31	(0.96,1.76)	0.09
	Concern about adverse events	30.5	1.52	(1.12, 2.05)	0.01
	Low knowledge	51.1	1.91	(1.43,2.58)	<0.0001

CI: Confidence Intervals

To investigate how different levels of knowledge regarding measles and its vaccination and varying degrees of concern about possible adverse events could influence uncompleted vaccination, respondents were categorized into four groups according to their levels of knowledge (cutoff points = the median values) and degree of concern. This logistic regression analysis was carried out using three dummy variables, namely, X1, X2 and X3. (Table 1) Results showed that the odds ratio of uncompleted measles vaccination was highest for Group 4 (i.e. "low knowledge" and high concern about adverse events), followed by Group 2 ("low knowledge" and low concern about adverse events), Group 3 ("high knowledge" and high concern about adverse effects), and Group 1 ("high knowledge" and low concern about adverse events). (Table 5) Among the 18-month-old group, measles vaccine coverage was 73.2%, the odds ratio may be inflated as an approximate value of the risk ratio.

**Table 5:** Factors related to uncompleted measles vaccination (multivariate analysis)

	18 month group			36 month group		
Variable	Odds Ratio	(95% CI)	P value	Odds Ratio	(95% CI)	P value
Mother's age (< 30 years)	1.29	(1.06, 1.59)	0.01	1.67	(1.15, 2.40)	0.007
Mother working	1.38	(1.00, 1.89)	0.05	1.75	(1.16, 2.66)	0.008
Not first born child	2.24	(1.84, 2.73)	<0.0001	2.38	(1.71, 3.34)	<0.0001
Interaction with other children	1.57	(1.13, 2.18)	0.008	1.90	(1.24, 2.89)	0.003
Presence of allergy	0.84	(0.66, 1.07)	0.17	1.23	(0.88, 1.71)	0.22
High knowledge, low concern (Group 1)	1.00			1.00		
Low knowledge, low concern (Group 2)	2.14	(1.67, 2.75)	<0.0001	2.08	(1.38, 3.19)	0.001
High knowledge, high concern (Group 3)	1.36	(0.97, 1.90)	0.07	1.69	(1.01, 2.79)	0.04
Low knowledge, high concern (Group 4)	3.67	(2.74, 4.93)	<0.0001	3.69	(2.30, 5.94)	<0.0001

CI: Confidence Intervals

## Discussion

Since 1978, measles vaccination has become part of routine vaccination schedules in Japan. Following the amendment to the Immunization Law in 1994, vaccination has become voluntary. According to this law, a single dose measles vaccine schedule is recommended for children over one year of age. Children are eligible to receive measles vaccination after 12 months following birth but not exceeding 90 months. In January 2004, a recommendation was issued that children should be vaccinated between 12 and 15 months of age, not between 12 and 24 months as it was previously. This change was proposed as a measure to prevent increased incidence of measles among non-vaccinated one-year-old children. Optional vaccination is available for individuals who are now older than the conventional age of vaccination. Due to low vaccine coverage, small or moderate outbreaks of measles are quite common in Japan. Although most infections occur in non-vaccinated young children around the age of one-year-old, cases of adult measles has also been on the rise [18-20]. Approximately 60% of reported measles cases in Japan involve people under the age of five years without a history of vaccination, and an increase in vaccination coverage at age one may be decreasing by approximately half, the total number of all measles patients [21].

To increase vaccine coverage against measles, an accurate estimate of the current rate of vaccine coverage is required. Presently, the coverage rate is calculated by dividing total number of persons vaccinated per year by total number of children between the ages of 12 and 24 months [11]. This formula does not accurately represent the actual number of children who have or have not been vaccinated against measles. In Japan, local governments are responsible for vaccination in their respective populations. Vaccination policy thus differs across different cities, towns and villages (namely, individual or mass vaccination, provided with or without charge, and vaccination age ranging from 12 to 18 months). Approximately 90% of local governments in Japan, including Kyoto City, provide vaccination to children who are older than one-year on an individual basis without charge [13]. According to the nation-wide survey conducted in 2000 (total number of persons vaccinated against measles per year over total number of persons who were going to receive vaccination per year), measles vaccine coverage in Japan is 81.4%, surpassing 80% for the first time since the survey initially began [13]. This survey's methodology, however, is problematic. The definition of denominator (total number of persons who were going to receive vaccination per year) differs across individual local governments. To obtain vaccine coverage is important for each local government.

Kyoto City experienced moderate-scale measles outbreaks in 1984, followed by smaller outbreaks in 1987, 1992 and 1997 at five-year intervals. The number of reported cases of measles at one point in 2001 was 205, with no reported cases of further spread. A further report identified that 44.4% of all measles cases occur in children ages two or below [22].

In the present survey, measles vaccine coverage, which was calculated by simply dividing the number of respondents whose children had been vaccinated by total number of respondents, was 73.2% among the 18-month-old group and 88.9% among the 36-month-old group. The percentage of children in Kyoto City that actually received health checkup was 90.6% among 18-month-olds and 85.7% among 36-month-olds. Because it can be assumed that vaccine coverage rate may be lower among children who do not receive health checkups, actual coverage rate may also be lower than that indicated in the present study.

Several previous surveys in Japan report vaccine coverage among 18-month children to be around 70% and that among 36-month children to be around 90% [23-25]. These findings are consistent with the present study. Several studies have also reported that factors related to a child-bearing environment, such as young motherhood, mother working, not being the first-born child, and attending nursery school, may contribute to low vaccine coverage [23-25]. Our findings agree. The present study also confirmed that factors such as concern about adverse events and low levels of knowledge were significantly associated with uncompleted measles vaccination. We checked for colinearity in the multivariate regression model. The correlation coefficients except among "mother working" and "interaction with other children" was 0.01–0.29. Although the correlation coefficients among "mother working" and "interaction with other children" was high (0.75 in 18-month group and 0.66 in 36-month group), these are different matters and we are interested in both of them. We put both of them into the final model, and got the result that we can interpret.

Lieu textitet al. examined the MMR vaccination history among 15-month-old children from affluent families and the factors that may delay vaccination. These factors were: having a large number of children, not having a regular doctor, not knowing when the shot was due, and not worrying about the risk of shots [26]. The present study showed that measles vaccine coverage was lowest among the group of children whose mothers were concerned about the adverse events of the vaccine without proper knowledge of measles and its vaccination (the coverage: was 18-month-old group: 61.9%, 36-month-old group: 85.0%). Even among mothers who indicated their concern about adverse events, the odds ratio of uncompleted measles vaccination was higher in those who had insufficient knowledge regarding measles and its vaccination. These results suggest that the level of knowledge is more highly correlated with immunization status than the level of concern about adverse events; this highlights the importance of promoting awareness among parents regarding measles vaccination. Effort is needed to improve benefit and risk communication [27]. Most important to vaccination is a balanced approach to the potential risks of natural infection, vaccine benefits, and adverse reactions to the vaccine. Given the potential risk of natural measles infection and the vaccine's efficiency and safety, the benefits are obvious [21].

As part of a campaign to raise awareness of measles vaccination in Japan among parents whose children are about to be enrolled in nursery schools, kindergartens and elementary schools, the Japanese Medical Association and the Japanese Association of Pediatrics have jointly launched a Vaccination Week for Children in March, 2004. During this Vaccination Week, consultation regarding measles vaccination was available to parents, and vaccination was carried out both on Saturday and Sunday.

## Conclusion

In order to promote measles vaccination, further consideration should be given to the following measures: to promote awareness about measles, the benefits of measles vaccine, and the possibility of adverse events from vaccination among parents, especially among those who are aged below 30 or those who have two or more children; to establish a system to enhance access to vaccination services by, for example, providing vaccination services at nights and on weekends, and; to encourage and support nursery school staff in promoting vaccination as well as in collecting vaccination history data about the children.

## Competing interests

The author(s) declare that they have no competing interests.

## Authors' contributions

TM conceived of the study and completed the analysis. TN supervised all aspects of its implementation and was in charge of its writing. SO and HI assisted with the study and analysis. All authors read and approved the final manuscript.

## Pre-publication history

The pre-publication history for this paper can be accessed here:


